# Reported Cases of Serotonin Syndrome in MDMA Users in FAERS Database

**DOI:** 10.3389/fpsyt.2021.824288

**Published:** 2022-01-24

**Authors:** Tigran Makunts, Lisa Jerome, Ruben Abagyan, Alberdina de Boer

**Affiliations:** ^1^MAPS Public Benefit Corporation, San Jose, CA, United States; ^2^Skaggs School of Pharmacy and Pharmaceutical Sciences, University of California, San Diego, San Diego, CA, United States

**Keywords:** serotonin syndrome, MDMA (3, 4- methylenedioxymethamphetamine), FAERS database, surveillance system, case reports [publication type]

## Abstract

3,4-Methylenedioxymethamphetamine (MDMA), is investigated as a treatment for post-traumatic stress disorder and other anxiety-related conditions in multiple placebo-controlled and open label studies. MDMA-assisted therapy is projected for approval by the United States Food and Drug Administration (FDA) and other regulatory agencies worldwide within the next few years. MDMA is a monoamine releaser and uptake inhibitor affecting serotonin, potentially increasing the risk of serotonin syndrome (SS). No instances of SS have occurred in clinical trials. The relatively small number of patients in controlled trials warranted a survey of FDA Adverse Event Reporting System data for the occurrence of SS in a larger database. We found 20 SS cases in people exposed to MDMA, all of which had also taken one or more substances with serotonergic properties in addition to MDMA, including amphetamines, stimulants, and opioids. There were no cases of SS associated with MDMA where MDMA was the sole reported compound taken.

## Introduction

In the European Union and the United States, 3,4-methylenedioxymethamphetamine (MDMA) is currently a schedule I controlled substance (Class A in the United Kingdom). The interest in MDMA use in psychiatry has solidified and is growing following publications of results from multiple controlled trials including a Phase 3 study for MDMA assisted therapy for post-traumatic stress disorder (PTSD) (1–4).

MDMA's psychoactive properties are due to multiple mechanisms that modulate monoamine neurotransmission, including release and reuptake of serotonin, dopamine and norepinephrine (5–8). Proposed therapeutic mechanisms of MDMA may include increased ability to confront upsetting memories, supporting fear-extinction learning and increased interpersonal closeness (9–11). Adverse events observed in controlled trials included transient hypertension, muscle tightness, decreased appetite, nausea, hyperhidrosis and feeling cold (1, 12).

Serotonin syndrome (SS) is a potentially life-threatening condition resulting from serotonergic over-activity at synapses of the central and peripheral nervous systems usually involving serotonergic medications (13). SS manifests itself through a range of mild to severe symptoms. Mild symptoms include akathisia and tremors, and severe symptoms include hyperthermia and muscular rigidity, which can be life-threatening (14).

Although not observed under controlled conditions, MDMA use beyond research settings has been associated with SS in case reports and toxicology studies (15–18). The vast majority of SS clinical case reports in published literature include a combination of two or more serotonergic agents including various classes of antidepressants, and other medications with serotonergic activity such as opioids (tramadol), antibiotics (linezolid), antihistamines (diphenhydramine), and atypical antipsychotics (19–24).

Given the high percentage of the PTSD population for whom serotonin modulating therapeutics are prescribed (25, 26) and the high prevalence of other PTSD comorbid conditions, including substance use (27), depression (28), anxiety (29), sleep (30), and pain disorders (31) treated by serotonergic drugs, further exploration of MDMA related Adverse Events (AE) reports from the drug safety surveillance database in the FDA Adverse Event Reporting System (FAERS) is warranted.

In this study, we evaluated individual cases listing MDMA use associated with SS and reported to FAERS through MedWatch (32). We evaluated reports for the presence of MDMA as the sole reported compound, and for the presence of any additional substances or medications, particularly those that might increase the risk of SS due to their inherent serotonergic activity.

## Methods

### FDA Adverse Event Reporting System

FAERS is an AE case repository for drugs and biologics reported to the FDA through MedWatch (32, 33). Cases include voluntary AE reports by consumers, healthcare professionals, legal representatives, and manufacturers.

FAERS was initially intended for post-marketing drug and biologic surveillance. However, it has historically included drugs pending approval and even schedule I controlled substances. Since there are no phase 4 trials for the latter, FAERS is an important source of safety data, as it provides meaningful safety signals which may help in diagnosing and mitigating illicit drug toxicity cases in the real world. Additionally, reporting use of illegal or unapproved substances to FAERS is important because they may often be the culprit of an adverse event as is often seen in polypharmacy cases.

### Combining and Normalizing Data Sets

Quarterly FAERS/AERS data sets were downloaded individually from the FDA's public repository and saved in a dollar-sign separated text format. Each quarterly dataset includes a data subset which refers to a specific variable or variables in the AE report (demographics, drug, indication, outcome, reaction, report source, therapy). The AE reports were recompiled using the case numbers common in each of the subsets. The study covered over 16 million reports from FAERS from September 2004 through June 2021. Because incomplete reporting and paucity of data did not allow a uniform format in all quarters/years, we standardized the data sets to create a consistent structure (34, 35) with blank tables replacing missing values. Unix/Linux code was used in data restructuring and manipulation. A total of 16,014,341 AE reports were obtained.

## Results

There were 1,143 AE reports which included MDMA in FAERS/AERS; 20 of the reports listing MDMA were reports of SS. Interestingly FAERS/AERS contained only one case of MDMA (reported as ecstasy) was identified as the sole responsible compound; a report of cardiomyopathy. Nineteen of the reports were submitted by healthcare professionals, while one report was submitted by the consumer (a voluntary report by an individual). There were no reports of SS where MDMA was identified as the sole responsible compound. The remainder of the MDMA AE reports (*n* = 1,142) included MDMA and at least one or more concomitant drug. The most common class of drugs reportedly taken along with MDMA in cases of SS were amphetamines (12 reports), followed by opioids (10 reports), benzodiazepines and sedative hypnotics (8 reports), cannabis or tetrahydrocannabinol (THC) (8 reports), selective serotonin reuptake inhibitors (SSRIs) (6 reports), monoamine oxidase inhibitors (MAOIs) (4 reports), 2nd generation antipsychotics (3 reports), cocaine (2 reports), alcohol (2 reports), ergot alkaloids (1 report), serotonin-norepinephrine reuptake inhibitors (SNRIs) (1 report), and ketamine (1 report) (Tables 1, 2).

**Table 1:** Individual cases of serotonin syndrome.

	**Age**	**Sex**	**Country**	**Concomitant medications**	**Adverse events**	**Outcome**	**Reported by**
1	24	M	Germany	PS-Clozapine SS-MDMA C-amisulpride C-zopiclone C-olanzapine C-lorazepam	Serotonin syndrome Convulsions Psychotic disorder	OT	MD
2	25	M	Turkey	PS-fentanyl SS-ephedrine SS-MDMA SS-ergot alkaloids SS-marijuana C-bupivacaine C-midazolam C-propofol C-vecuronium	Serotonin syndrome	HO	MD
3^*^(2)	20	F	UK	PS-oxycodone SS-MDMA	Serotonin Syndrome	DE	CN
4	24	M	Australia	PS-dextroamphetamine and amphetamine salts SS-MDMA SS-methamphetamine HCl SS-moclobemide	Serotonin Syndrome	DE	Other HP
5	45	M	Australia	PS-dextroamphetamine and amphetamine salts SS-MDMA	Serotonin syndrome Drug interaction Drug toxicity	DE	Other HP
6	31	M	Australia	PS-dextroamphetamine and amphetamine salts SS-MDMA SS-methamphetamine HCl SS-moclobemide SS-THC	Serotonin syndrome	DE	Other HP
7	25	F	Australia	PS-dextroamphetamine and amphetamine salts SS-MDMA SS-methamphetamine HCl	Serotonin syndrome	DE	Other HP
8	24	M	US	PS-Lithium carbonate SS-MDMA SS-fentanyl SS-midazolam SS-phenelzine SS-propofol SS-suxamethonium (succinylcholine)	Blood CPK increased Clonus Hyperhidrosis Ileus Loss of consciousness Muscle twitching Mydriasis Myoclonus Nystagmus Serotonin syndrome Tachypnea	HO, OT	Other HP
9^*^(2)	16	M	US	PS-APAP hydrocodone SS-amphetamine SS-cocaine SS-marijuana SS-MDMA SS-methamphetamine HCl	Disseminated intravascular coagulation Hepatorenal failure Hyperthermia malignant Hypoglycemia Hypoxic ischemic encephalopathy Intentional drug misuse Multi organ failure Multiple drug Overdose intentional Rhabdomyolysis Serotonin syndrome Shock	DE	MD
10^*^(2)	28	M	Australia	PS-sertraline I-ethanol I-methamphetamine I-MDMA I-THC	Serotonin syndrome Back injury Head injury Road traffic accident	DE, OT	Other HP
11	25	M	Australia	PS-sertraline SS-MDMA SS-Cocaine C-St John's wort	Abdominal pain upper Aggression Alanine aminotransferase increased Aspartate aminotransferase increased Blood potassium decreased Blood pressure increased Decreased appetite Disorientation Drug abuse Electrocardiogram qt prolonged Heart rate increased Muscle rigidity Nausea Oxygen saturation decreased Serotonin syndrome Sinus tachycardia Vomiting Weight decreased	OT	Other HP, Literature
12	32	M	Australia	PS-alprazolam C-diazepam C-MDMA C-Methadone C-methamphetamine	Serotonin syndrome Toxicity to various agents	DE, HO	MD
13	30	F	Australia	PS-diazepam SS-MDMA SS-methamphetamine HCl SS-nordiazepam SS-THC SS-Tramadol	Serotonin syndrome Toxicity to various agents	DE, OT	Other HP
14	21	M	Australia	PS-diazepam SS-MDMA SS-methamphetamine HCl SS-amphetamine SS-codeine SS-fluoxetine SS-ketamine SS-morphine SS-Sertraline SS-temazepam	Serotonin syndrome Toxicity to various agents Pulmonary edema	DE, OT	Other HP
15	28	M	Australia	PS-citalopram SS-MDMA SS- 7-aminoflunitrazepam SS-amphetamine SS-codeine SS-Methamphetamine SS-morphine SS-THC	Serotonin syndrome Toxicity to various agents	DE, OT	Other HP
16	34	M	Australia	PS-Citalopram SS-MDMA SS- 6-monoacetylmorphine SS-ethanol	Hepatic steatosis Prostatitis Serotonin Syndrome Toxicity to various agents	DE, OT	Other HP
17	16	M	US	PS- methamphetamine HCl SS-MDMA	Accidental death Disseminated intravascular coagulation Encephalopathy Hemodialysis Hepatorenal failure Hyperthermia malignant Hypoglycemia Hypotension Hypoxic ischemic encephalopathy Multi organ failure Multiple drug overdose Muscle rigidity Renal failure Rhabdomyolysis Serotonin syndrome Shock Tremor Unresponsive to stimuli	DE	MD
18^*^(2)	41	M	US	PS-desvenlafaxine SS-Risperidone I-MDMA	Acute kidney injury Aggression Blood sodium decreased Drug interaction Hemodialysis Rhabdomyolysis Serotonin syndrome	HO, OT	Other HP
19	unknown		France	PS-fentanyl SS-buprenorphine SS-cannabis SS-cocaine SS-codeine SS-MDMA SS-heroin SS-hydromoprhone SS-methadone SS-morphine SS-Oxycodone SS-remifentanyl	Serotonin syndrome	OT	Other HP
20^*^(9)	17	F	France	PS-fluoxetine SS-aripiprazole SS-diazepam SS-olanzapine SS-amphetamine SS-cannabis SS-MDMA	Serotonin syndrome Somnolence Tachycardia Miosis Agitation Intentional overdose	HO	Consumer Pharmacist Other HP

*PS, primary suspect; SS, secondary suspect; I, interacting C-concomitant; HO, hospitalization; C, concomitant; OT, Other Serious (Important Medical Event); DE, death; CN, consumer; HP, health professional; MD, doctor of medicine*.

* *(), number of duplicates*.

**Table 2:** Psychoactive concomitant drugs, summarized by class, in serotonin syndrome/MDMA cases.

	**MDMA**	**Amphetamines**	**MAOI**	**SSRI**	**SNRI**	**2nd gen antipsychotic**	**Cannabis or THC**	**Cocaine**	**Benzodiazepines and GABA modulators**	**Opioids**	**Alcohol**	**Ergot alkaloids, triptans**	**Ketamine**
1	*					***			**				
2	*						*		*	*		*	
3	*									*			
4	*	**	*										
5	*	*											
6	*	**	*				*						
7	*	**											
8	*		*						*	*			
9	*	**					*	*		*			
10	*	*		*			*				*		
11	*		*	*				*					
12	*	*							**	*			
13	*	*					*		**	*			
14	*	**		**					**	**			*
15	*	**		*			*		*	**			
16	*			*						*	*		
17	*	*											
18	*				*	*							
19	*						*	*		*********			
20	*	*		*		**	*		*				

Number of * corresponds to the number of drugs in the listed medication class included in the report.

Seventeen out of 20 cases included two or more concomitant psychoactive substances. The Pubmed library was queried (using SS and MDMA, midomafetamine, 3,4-methylenedioxymethamphetamine, 3,4-methylenedioxy-methamphetamine, molly, and ecstasy terms), to confirm whether any of the presented cases were present in the literature, and no published reports were found, possibly due the MDMA being designated as not the primary suspect in all the cases.

## Discussion

In this study, we evaluated SS cases associated with MDMA use reported to the FDA using the FAERS system. We found no reports of SS in cases where MDMA was the sole reported drug, which confirmed the observed lack of SS in clinical trials. Additionally, we observed a limited number of 20 cases of SS associated with use of MDMA reported in the last ~17 years. All of those cases listed additional serotonergic psychoactive drugs, with 85% of the reports including at least two other drugs with serotonergic properties. It should be noted that none of the reports considered MDMA the “primary suspect” (PS) of the AE adjudicated by the reporter. There is a possibility that MDMA contributed to the AE profile through CYP2D6-mediated drug-drug interaction (36, 37). Considering the large number of people who report using ecstasy, estimated by the United Nations Office of Drugs and Crime to be nearly twenty million people (38), the number of MDMA FAERS/AERS reports was surprisingly low.

### Study Limitations

Since reporting to FAERS is mostly voluntary, apart from spontaneous reports forwarded from the manufacturers/authorization holders, the data set represents only a subset of actual cases and therefore the FAERS case frequencies should not be confused with absolute population incidences. Most of the cases are not clinically assessed for causality, and detailed case narratives are not provided to maintain patient privacy and protected health information. There was no consistent means for reporters to provide information on drug identification or detection.

Nineteen out of the 20 presented case reports were submitted by healthcare professionals (*Form*-3500), with the reported outcome of either death or hospitalization, wherein it is standard clinical practice to administer drug tests to identify cause of toxicity. However, since manufacture and distribution of MDMA is not regulated, it is still uncertain whether material included in the cases could be confirmed as MDMA or MDMA laced with another compound. SSRIs' protective effects on the pharmacodynamic effects of MDMA have been well-documented (39–42). Although SSRis were present in six of the presented cases, all of the reports listed additional substances as well. Due to the low number of MDMA+SSRI SS cases in FAERS, this relationship was challenging to quantify.

## Conclusion

In summary, reported use of MDMA as the sole administered drug produced no reports of SS in the FAERS system; it was far more common for this syndrome to arise when MDMA was reportedly combined with an additional substance, including psychostimulants, opioids, and antidepressants. In clinical trials of MDMA-assisted therapy, participants are tapered off serotonergic drugs prior to administration of MDMA. The current findings in the FAERS system are in line with the failure of clinical trials where MDMA is investigated in conjunction with therapy to report SS.

## Data Availability Statement

The datasets presented in this study can be found in online repositories. The names of the repository/repositories and accession number(s) can be found below: https://www.fda.gov/drugs/questions-and-answers-fdas-adverse-event-reporting-system-faers/fda-adverse-event-reporting-system-faers-latest-quarterly-data-files.

## Ethics Statement

Ethical review and approval was not required for the study on human participants in accordance with the local legislation and institutional requirements. Written informed consent from the participants' legal guardian/next of kin was not required to participate in this study in accordance with the national legislation and the institutional requirements.

## Author Contributions

TM performed the research. TM, LJ, RA, and AB designed the study, drafted the manuscript, and reviewed the final version. RA processed the data sets. AB supervised the study. All authors contributed to the article and approved the submitted version.

## Funding

The study was funded by MAPS Public Benefit Corporation and in part by Skaggs School of Pharmacy and Pharmaceutical Sciences, UC San Diego Health.

## Conflict of Interest

The authors declare that the research was conducted in the absence of any commercial or financial relationships that could be construed as a potential conflict of interest.

## Publisher's Note

All claims expressed in this article are solely those of the authors and do not necessarily represent those of their affiliated organizations, or those of the publisher, the editors and the reviewers. Any product that may be evaluated in this article, or claim that may be made by its manufacturer, is not guaranteed or endorsed by the publisher.
